# Supplementary material for: Parallels, differences and lessons: a comparison of the management of foot-and-mouth disease and COVID-19 using UK 2001/2020 as points of reference
Source: Proc Biol Sci. 2020 Nov 4;287(1938):20200906. doi: 10.1098/rspb.2020.0906 (PMC7735262; doi:10.1098/rspb.2020.0906)
Supplement: Supplementary information on the transmission and control of FMD in relation to COVID19 [file rspb20200906supp1.docx]

Supplementary material

**Parallels, differences and lessons: a comparison of the management of foot-and-mouth disease and COVID19 using UK 2001/2020 as points of reference**

Keith Sumption, Theodore J.D. Knight-Jones, Melissa McLaws & David J Paton

DOI: 10.1098/rspb. RSPB-2020-0906 Compiled June 2020

*Comparative Transmission*

For FMD virus, the most common site of primary infection is the upper respiratory tract or oropharynx, after which a viraemia seeds virus throughout the body and high levels of virus are shed from ruptured vesicles and in many secretions and excretions, including breath. This shedding can be quite prolonged, but direct cattle-to cattle transmission has now been shown to be efficient during only a much narrower window (~1.5 days) shortly before and after disease begins [1]. Fomites and infected animal products (meat, milk, offals) can also transmit the infection and are an important source of disease incursion into new areas and between countries. Long distance airborne spread, although rare, was probably significant at the start of the 1967 and 2001 epidemics in the UK, and in the small 1981 UK/Jersey outbreak [2]. In the UK2001 epidemic, the source of infection for ~80% of farms (infected premises) could not be identified and was attributed to local spread by unknown routes after imposition of livestock standstills [3].

Contact tracing in FMD control to identify “dangerous contacts” is logistically challenging given the multiplicity of transmission opportunities and especially when significant time has elapsed between the release of infection into a previously free population and first confirmed case. Retrospective sequencing studies confirmed the difficulty of establishing indirect transmission routes from conventional epidemiological investigations [4]. Epidemiological investigations, latterly aided by full-genome sequencing, have elucidated many chains of “community transmission” of FMDV in the 2001 epidemic. The lower mutation rate in coronaviruses compared to FMDV may reduce the power of genetic comparisons to resolve transmission pathways.

Like FMDV, COVID19 is also highly contagious and thought to be transmitted by a combination of respiratory droplets and fomites [5]. However, as a new disease, there are many vital information gaps and understanding the determinants of efficient transmission will be important for targeting control measures (Table S1). The opportunities for disseminating infection through direct and indirect contacts are likely to be much greater within the human population than for livestock, as livestock in developed countries are mainly raised in semi-isolated epidemiological units (farms) with occasional, documented movements and mixing. However, group sizes for livestock are often hundreds of times larger than human households.

*R0 and mortality rates*

Reproduction number (R0) estimates for FMDV range from 2-70 for within-herd transmission depending on species susceptibility and husbandry conditions [6–10]. However, as livestock typically live in close contact with their herd-mates, but in limited contact with other herds, between-herd transmission is lower and controlling this is key to controlling outbreaks [11]. For the UK 2001, Ferguson et al., 2001 [12] estimated the initial between-herd R0 to be 4.5, dropping to about 1.5 by mid-March[13], with broadly similar estimates made for outbreaks in other countries [14,15]. Unlike SARS-CoV-2, transmission among livestock has been assessed in experimental challenge studies [16].

R0 estimates for COVID-19 have typically ranged from 2-4, with greater values often occurring in more densely populated areas (USA national average 2.8 [1.9 Nebraska - 4.5 New York] [17], Europe average 3.87 [3.01-4.66] [18,19], 2.0-3.5 in Wuhan [20], possible much higher [21], and China is 2.38 [95% credible interval (CI): 2.03−2.77 [22].

A major part of the COVID-19 impact arises from its severity, with a crude mortality ratio (the number of reported deaths divided by the reported cases) of 3-4%, compared to 0.1% for seasonal flu [23]. Fatality from FMD is similar (1-5%) in adults, depending on the virus strain and the host species and breed, but can sometimes be much higher in young animals [24] and in some wildlife species [25]. However, compared to other livestock diseases this is often considered a low mortality rate, and so the greatest impact of FMD arises from the high incidence and restrictions on trade [26].

*The spread of O PanAsia to involve the UK in 2001*

The spread of the O PanAsia strain from South Asia into South-East Asia in the 1990s, from where China and neighbouring countries were affected in 1998-99, preceded entry into previously free countries (Republic of Korea, Japan), even reaching South Africa, where it was controlled, in late 2000. The multiple routes of entry to these countries suggested a higher than normal “source prevalence” and risk to FMD free countries and this led to the warnings issued by the European Commission for the Control of FMD (EuFMD) in late 2000. The source of the UK FMD 2001 virus incursion is thought to be from illegally imported meat [27], highlighting the difficulty to reduce risk by border controls alone and a need to reduce risk at source. This is best achieved through strong national control programmes as well as regional and international cooperation, that minimises disruption to trade and economies and avoids creating incentives for hiding outbreaks or illegal movements and trade.

Controlling FMD transmission with "lockdowns"

Animal movements were severely limited from late February 2001 and, given that many animal owners had rented grazing or animals on multiple, discontinuous parcels of land, issues of feed supply to these were intense and even added to the problem of clinical disease recognition as sheep developed “starvation” lesions in the mouth resembling FMD. Large numbers of animals had to be euthanased on welfare grounds because they were unable to access suitable grazing. Livestock farms attempted to self-isolate, but with little recent experience of the levels needed to prevent FMD, the efforts were variable and at best hard and expensive to maintain in winter conditions. Nationwide footpath closures were imposed 4 days after the national animal movement standstill, and resulted in serious impact on leisure and tourism, and perhaps wellbeing [28,29]. Similarly, for COVID19, people were unable to return home from abroad and there has been a massive economic impact across the economy. The requirement for people to stay at home has led to questions about how readily the virus can be spread in outdoor spaces so long as people remain dispersed, especially in sparsely populated areas. Strikingly, the epidemics of COVID19 and FMD2001 were both taking off in the UK in late February but only with FMD was the Cheltenham horse racing festival cancelled (even though neither horses nor people are FMD susceptible).

Although animal movement control is regularly applied at single farm levels, once extended to national scale the impacts place enormous cost on the livestock industry and beyond. In the UK, farmers receive compensation for animals that are culled due to FMD, but consequential losses resulting from restrictions are not available. This can create a perverse incentive to have animals become infected. The restrictions applied to control COVID19 are unprecedented in the recent control of human diseases, affecting all economic activities, in cities and the country. Unlike FMD, publically funded schemes to support incomes of a wide range of sectors have been made with COVID19, to help ensure public acceptance of isolation measures, but there will still be many that lose out.

The epidemic lasted until the end of September 2001 (32 weeks), but not until 22 January 2002 was the United Kingdom re-instated on the OIE-list of countries free of FMD, and on 5 February 2002 the European Commission lifted remaining meat and animal export restrictions. The direct cost to the public and private sectors was estimated at over £3 billion and £5 billion respectively, with a net economic effect of the outbreak of 0.2% GDP [30,31]. By the time the disease had been eradicated in September 2001, animals on more than 9,500 farms (~5-8% of susceptible holdings) had been slaughtered out. The actual number of individual animal FMD cases cannot be known as individual animals were not tested before slaughter. In contrast to the 1950s, when the ratio of secondary cases to primaries was circa 2:1; with the FMD2001, considering the holdings infected before confirmation as primaries, the final ratio was over 35, though with some of these primaries, initial containment had succeeded.

The evolution of approaches to COVID19 control differs from FMD2001 in the UK, where containing the infection by stamping-out measures was the tactic through-out. In the Netherlands, in contrast, emergency vaccination was used on all farms in the FMD infected zone. Vaccination “flattened the curve” of logistical actions needed on farms and allowed critical resources to be built up to allow an orderly disposal of vaccinated animals. In 2001, it can be argued the UK control strategy aimed at *shortening* the curve, but the message did not resonate with the general public; the response to backlogs of undisposed carcasses was to increase speed of slaughter targets and bring in the military to manage the logistics of mass burials.

## Shortening the long tail: belatedly learning the importance of biosecurity in animal epidemic control

The unprecedented culling programme in March and April 2001 had its impact on the epidemic which peaked in early April. However the tail of the epidemic was long and very costly, and in the decision not to use vaccination, every new case brought a risk of the epidemic re-igniting. The last, lingering foci were addressed and finally extinguished by a much tighter application of a new tool - “surveillant biosecurity procedures” [32], in 3 designated areas (“blue boxes”), whose success was considered to be the policy change of combining surveillance with biosecurity procedures in order to achieve greater isolation of every farm holding. It is astonishing that it was so late in the epidemic before this was introduced, and this may reflect the complete behavioural change needed at every level, for which veterinarians and farmers in 2001 had little prior acceptance or practice. Far more attention has been given to behavioral aspects of biosecurity in COVID19, perhaps re-inforced by images from other countries of compliance. Better public engagement has been coupled with community re-inforcement of the messages, than was initially applied to FMD, where there was much less of an inter-agency approach to achieving between-farm isolation. The use of drones in 2020 may be considered a form of "surveillant biosecurity" that may be more acceptable if applied to animal movements than to the human population.

*The battle for public opinion and support for control measures*

After the weeks of animal culling height of the FMD2001, the image of a calf (“Phoenix”) that had survived 5 days after "euthanasia" was the national good news story long sought [33], a grateful Government combined this on the 25th April with declaring a relaxing of restrictions as the battle was “being won”. The “Phoenix” moment of 2020 was perhaps the image of Boris Johnson, released from hospital on the 12th April after 3 days of intensive care, an image of a fight-back and perhaps the start of national recovery.

In 2001, social networks of farmers, activists and concerned public rapidly formed to assist each other and led an intense debate about control measures. Many initiated legal proceedings against the “area-based” culling of their herds; some imported unvalidated POC tests and taught each other via the internet to test their animals to assist their claims to be free of infection. A Judicial Review of Government Policy, which would have halted the extended ring culls in favour of vaccination was successful in its initial reading, and was only discontinued when the Prime Minister of the time (Tony Blair) signaled his intention to pursue vaccination and had ordered the vaccine. The case for vaccination as an adjunct measure was made by a wide body of opinion including the Soil Association (Organic certification body), but equally vigorously opposed by the National Farmers Union who considered the long term economic impacts would be worse if vaccination was adopted. The COVID19 crisis does not so far appear to have been faced with similar Judicial Reviews, or anti-vaccination camps, though analogous campaigns against COVID 19 control measures may yet emerge as more and more feel their economic impact, or face obligations to be vaccinated.

**Table S1. FMDV/SARS-CoV-2 comparative transmission parameters. Completed June 2020.**

| **Virus transmission parameter** | **FMD^1^** | **SARS-CoV-2** |
| --- | --- | --- |
| Key wildlife reservoir | African buffalo for SAT^2^ serotypes[25,34] | Bats[35] |
| Incubation period (range) | 1-14d[36,37] | 1-14d[38] |
| Incubation period (most common) | 2-6d[36,37] | 5-6d[38,39] |
| Close contact respiratory droplet transmission | Yes[40] | Yes[41,42] |
| Preclinical respiratory shedding | 1-2d^3^ [43] | 1-2d[35] |
| Peak airborne emissions/day | pigs~10^6^TCID_50_[44] | ? |
| Duration of oronasal shedding | 5-8d^4^[45] | 8-11d[46] |
| Range of excretions and secretions in which virus shed | All secretions and excretions[47] | Saliva, resp. tract secretions, faeces[48] |
| Minimum inspired infectious dose | 10 TCID_50­­_[49] | ? |
| Fomite transmission | Yes[45] | Yes[50,51] |
| Entry via respiratory tract | Yes[45] | Yes[50] |
| Main portal of entry | Pharynx[52] | ? |
| Entry via mucosal/epidermal abrasions | Yes[53] | ? |
| Entry via ingestion (e.g. of animal products) | Yes^4^[49] | ?[54] |
| Long distance respiratory aerosol transmission | Possible[2] | ? |
| Duration of viraemia | 4-5d^5^ [36,37] | ? |
| R_0_ | >10[9,55] | Often 2-3[17,19–22] |
| First antibody detection after onset of clinical signs | 3-4d[56] | 6-15d[57] |
| Virus persistence beyond 28d | Up to 3.5 yrs[34] | Up to 37d[58] |
|  |  |  |
| ^1^ Inter-species variation exists | | |
| ^2^ Southern African Territories serotypes | | |
| ^3^ Sheep only, in cattle virus may be shed in milk ≤4 days before onset of vesicular lesions[59] | | |
| ^4^ But cattle require 100,000 higher dose than for respiratory infection[49] | | |
| ^5^ From a small case study | | |
| ^§^ Between animals (usually lower between herds) | | |

**Table S2. Similarities and differences in control of FMD 2001 and COVID 19 in the UK***

| **Feature** | **Common** | **FMD 2001 Specific** | **COVID 19 Specific** |
| --- | --- | --- | --- |
| Overall aims of control measures |  | For FMD-free countries such as UK 2001: rapid eradication to recover trade and promote long-term welfare and efficiency of production.  [A range of priorities in other parts of the world, particularly where FMD is endemic.] | In UK and some other countries: containment to save lives and mitigate wider impacts until vaccination is available  [In other countries, aims have ranged from mainly letting the disease run to eradication of the infection] |
| Spread of disease | Importance of respiratory droplets and fomites in virus transmission | Other important routes of transmission, such as ingestion of animal products.  [Long-term wildlife reservoir (Syncerus caffer) in parts of Africa.] | Original wildlife reservoir |
|  |  | Multiple livestock hosts mostly kept in herds, with differences in husbandry, susceptibility, virus shedding and morbidity. | Global pandemic involving one principal host - man |
| Preparedness | Cognitive biases in preparedness and threat assessment | Known disease | New disease |
|  |  | Pre-existing measures to prevent international spread | No pre-existing measures to prevent international spread |
| Management | Responses delayed until many cases | Point-source introduction | Multifocal introduction |
|  | Quarantine, lockdown and biosecurity based control measures | Culling and vaccination options | Culling and vaccination not options |
|  | Overwhelmed testing and response capacity | Control achieved largely without virological testing to confirm clinical diagnosis, but serology used to identify undisclosed foci | Virological testing deemed critical for contact tracing once lockdown eased |
|  | Different interpretations of what science is telling us |  |  |
|  | Mathematical model outputs drove more aggressive control response |  |  |
| Recovery and long-term management | Need to deal with asymmetry of disease status of different countries | Time to reflect on FMD lessons | COVID 19 currently unfolding |

* Some comparative features of control in other countries added in parenthesis []

**Supplement references**

1. Charleston B *et al.* 2011 Relationship Between Clinical Signs and Transmission of an Infectious Disease and the Implications for Control. *Science (80-. ).* **332**, 726–729. (doi:10.1126/science.1199884)

2. Donaldson AI, Gloster J, Harvey LDJ, Deans DH. 1982 Use of predictive models to forecast and analyse airborne spread during the foot-and-mouth disease outbreaks in Brittany, Jersey and the Isle of Wight in 1981. *Vet. Rec.* **110**, 53–57.

3. Gibbens JC, Sharpe CE, Wilesmith JW, Mansley LM, Michalopoulou E, Ryan JB, Hudson M. 2001 Descriptive epidemiology of the 2001 foot-and-mouth disease epidemic in Great Britain: the first five months. *Vet Rec* **149**, 729–743.

4. Cottam EM, Haydon DT, Paton DJ, Gloster J, Wilesmith JW, Ferris NP, Hutchings GH, King DP. 2006 Molecular Epidemiology of the Foot-and-Mouth Disease Virus Outbreak in the United Kingdom in 2001. *J. Virol.* **80**, 11274–11282. (doi:10.1128/JVI.01236-06)

5. Harapan H *et al.* 2020 Coronavirus disease 2019 (COVID-19): A literature review. *J. Infect. Public Health*. **13**, 667–673. (doi:10.1016/j.jiph.2020.03.019)

6. Woolhouse MEJ, Haydon DT, Pearson A, Kitching RPP. 1996 Failure of vaccination to prevent outbreaks of foot and mouth disease. *Epidemiol Infect* **116**, 363–371.

7. Tadesse B, Molla W, Mengsitu A, Jemberu WT. 2019 Transmission dynamics of foot and mouth disease in selected outbreak areas of northwest Ethiopia. *Epidemiol. Infect.* **147**. (doi:10.1017/S0950268819000803)

8. Belayneh N, Molla W, Mesfine M, Jemberu WT. 2019 Modeling the transmission dynamics of foot and mouth disease in Amhara region, Ethiopia. *Prev. Vet. Med.* (doi:10.1016/j.prevetmed.2019.04.002)

9. Chis Ster I, Dodd PJ, Ferguson NM. 2012 Within-farm transmission dynamics of foot and mouth disease as revealed by the 2001 epidemic in Great Britain. *Epidemics* **4**, 158–169. (doi:S1755-4365(12)00034-5 [pii]10.1016/j.epidem.2012.07.002 [doi])

10. Hayer SS *et al.* 2018 Foot-and-mouth disease virus transmission dynamics and persistence in a herd of vaccinated dairy cattle in India. *Transbound. Emerg. Dis.* **65**, e404–e415. (doi:10.1111/tbed.12774)

11. Tildesley MJ, Keeling MJ. 2009 Is R0 a good predictor of final epidemic size: Foot-and-mouth disease in the UK. *J. Theor. Biol.* **258**, 623–629. (doi:10.1016/j.jtbi.2009.02.019)

12. Ferguson NM, Donnelly CA, Anderson RM. 2001 The Foot-and-Mouth Epidemic in Great Britain: Pattern of Spread and Impact of Interventions. *Science (80-. ).* **292**, 1155–1160. (doi:10.1126/science.1061020)

13. Haydon DT, Chase-Topping M, Shaw DJ, Matthews L, Friar JK, Wilesmith J, Woolhouse ME. 2003 The construction and analysis of epidemic trees with reference to the 2001 UK foot-and-mouth outbreak. *Proc Biol Sci* **270**, 121–127. (doi:10.1098/rspb.2002.2191)

14. Perez AM, Ward MP, Carpenter TE. 2004 Control of a foot-and-mouth disease epidemic in Argentina. *Prev. Vet. Med.* **65**, 217–226. (doi:10.1016/j.prevetmed.2004.08.002)

15. Chowell G, Viboud C, Simonsen L, Moghadas SM. 2016 Characterizing the reproduction number of epidemics with early subexponential growth dynamics. *J. R. Soc. Interface* **13**. (doi:10.1098/rsif.2016.0659)

16. Hagenaars TJ, Dekker A, de Jong MC, Eble PL. 2011 Estimation of foot and mouth disease transmission parameters, using outbreak data and transmission experiments. *Rev Sci Tech* **30**, 467–477.

17. Juliette H, Unwin T, Mishra S, Bradley VC, et al. 2020 Imperial College London Report 23: State-level tracking of COVID-19 in the United States - VERSION 2 (28-05-2020). (doi:doi.org/10.25561/79231)

18. Flaxman S *et al.* 2020 Estimating the effects of non-pharmaceutical interventions on COVID-19 in Europe. *Nature* **Online**. (doi:10.1038/s41586-020-2405-7)

19. Flaxman S, Mishra S, Gandy A, et al. 2020 Imperial College London Report 13: Estimating the number of infections and the impact of non-pharmaceutical interventions on COVID-19 in 11 European countries. Imperial College London (30-03-2020). (doi:doi.org/10.25561/79231)

20. Zhang J *et al.* 2020 Age profile of susceptibility, mixing, and social distancing shape the dynamics of the novel coronavirus disease 2019 outbreak in China. *medRxiv Prepr.* (doi:10.1101/2020.03.19.20039107)

21. Sanche S, Lin YT, Xu C, Romero-Severson E, Hengartner N, Ke R. 2020 High Contagiousness and Rapid Spread of Severe Acute Respiratory Syndrome Coronavirus 2. *Emerg. Infect. Dis.* **26**. (doi:10.3201/eid2607.200282)

22. Li R, Pei S, Chen B, Song Y, Zhang T, Yang W, Shaman J. 2020 Substantial undocumented infection facilitates the rapid dissemination of novel coronavirus (SARS-CoV2). *Science* **368**, 489–493. (doi:10.1126/science.abb3221)

23. WHO. 2020 Coronavirus disease 2019 (COVID-19) Situation Report – 46. Available at https://www.who.int/docs/default-source/coronaviruse/situation-reports/20200306-sitrep-46-covid-19.pdf?sfvrsn=96b04adf_4 Accessed 15 June 2020.

24. Iowa State University, OIE. 2007 Disease card -Foot and Mouth Disease. Available at: http://www.cfsph.iastate.edu/Factsheets/pdfs/foot_and_mouth_disease.pdf Accessed 15 June 2020.

25. Weaver G V, Domenech J, Thiermann AR, Karesh WB. 2013 Foot and mouth disease: a look from the wild side. *J Wildl Dis* **49**, 759–785. (doi:10.7589/2012-11-276 [doi])

26. Knight-Jones TJD, Rushton J. 2013 The economic impacts of foot and mouth disease - what are they, how big are they and where do they occur? *Prev Vet Med* **112**, 161–173. (doi:10.1016/j.prevetmed.2013.07.013)

27. Scudamore JM, Harris DM. 2002 Control of foot and mouth disease: Lessons from the experience of the outbreak in Great Britain in 2001. *OIE Rev. Sci. Tech.* **21**, 699–710. (doi:10.20506/rst.21.3.1351)

28. NAO Report. 2002 The 2001 Outbreak of Foot and Mouth Disease (full report) pp138. Report by the Comptroller and Auditor General, 21 June 2002. Available at https://www.nao.org.uk/wp-content/uploads/2002/06/0102939.pdf Accessed 14 June 2020. *HC 939 2001-2002*

29. Anderson I. 2002 UK Gov Foot and Mouth Disease 2001: Lessons to be Learned Inquiry Report. Available at: https://webarchive.nationalarchives.gov.uk/20100702233845/http://archive.cabinetoffice.gov.uk/fmd/fmd_report/report/index.htm Accessed 15 June 2020.

30. Thompson D, Muriel P, Russell D, Osborne P, Bromley A, Rowland M, Creigh-Tyte S, Brown C. 2002 Economic costs of the foot and mouth disease outbreak in the United Kingdom in 2001. *Rev. Sci. Tech.* **21**, 675–87.

31. Sobrino F, Domingo E. 2001 Foot-and-mouth disease in Europe. FMD is economically the most important disease of farm animals. Its re-emergence in Europe is likely to have consequences that go beyond severe alterations of livestock production and trade. *EMBO Rep.* **2**, 459–461. (doi:10.1093/embo-reports/kve122)

32. Donaldson A, Wood D. 2004 Surveilling Strange Materialities: Categorisation in the Evolving Geographies of FMD Biosecurity. *Environ. Plan. D Soc. Sp.* **22**, 373–391. (doi:10.1068/d334t)

33. The Guardian. 2001 Phoenix is a ‘ray of light’ for farming industry. Available at https://www.theguardian.com/uk/2001/apr/26/footandmouth1. Accessed 15 June 2020. 26 April.

34. Hedger RS. 1972 Foot-and-mouth disease and the African buffalo (Syncerus caffer). *J. Comp. Pathol.* **82**, 19–28. (doi:10.1016/0021-9975(72)90022-9)

35. WHO. 2020 Report of the WHO-China Joint Mission on Coronavirus Disease 2019 (COVID-19) 16-24 February 2020 www.who.int/docs/default-source/coronaviruse/who-china-joint-mission-on-covid-19-final-report.pdf Accessed 06 June 2020.

36. Alexandersen S, Quan M, Murphy C, Knight J, Zhang Z. 2003 Studies of quantitative parameters of virus excretion and transmission in pigs and cattle experimentally infected with foot-and-mouth disease virus. *J. Comp. Pathol.* **129**, 268–282. (doi:10.1016/S0021-9975(03)00045-8)

37. Alexandersen S, Zhang Z, Reid SM, Hutchings GH, Donaldson AI. 2002 Quantities of infectious virus and viral RNA recovered from sheep and cattle experimentally infected with foot-and-mouth disease virus O UK 2001. *J. Gen. Virol.* **83**, 1915–1923. (doi:10.1099/0022-1317-83-8-1915)

38. WHO. 2020 Coronavirus disease 2019 (COVID-19) Situation Report – 73 www.who.int/docs/default-source/coronaviruse/situation-reports/20200402-sitrep-73-covid-19.pdf?sfvrsn=5ae25bc7_4#:~:text=The%20incubation%20period%20for%20COVID,occur%20before%20symptom%20onset. - .

39. ECDC. 2020 Guidance for discharge and ending isolation in the context of widespread community transmission of COVID-19 – first update. https://www.ecdc.europa.eu/sites/default/files/documents/covid-19-guidance-discharge-and-ending-isolation-first%20update.pdf Access.

40. Alexandersen S, Zhang Z, Donaldson AI, Garland AJM. 2003 The Pathogenesis and Diagnosis of Foot-and-Mouth Disease. *J. Comp. Pathol.* **129**, 1–36. (doi:10.1016/s0021-9975(03)00041-0)

41. Liu J *et al.* 2020 Community Transmission of Severe Acute Respiratory Syndrome Coronavirus 2, Shenzhen, China, 2020. *Emerg. Infect. Dis.* **26**. (doi:10.3201/eid2606.200239)

42. Li Q *et al.* 2020 Early Transmission Dynamics in Wuhan, China, of Novel Coronavirus–Infected Pneumonia. *N. Engl. J. Med.* **382**, 1199–1207. (doi:10.1056/NEJMoa2001316)

43. Sellers RF, Parker J. 1969 Airborne excretion of foot-and-mouth disease virus. *J. Hyg.* **67**, 671–677.

44. Alexandersen S, Donaldson AI. 2002 Further studies to quantify the dose of natural aerosols of foot-and-mouth disease virus for pigs. *Epidemiol. Infect.* **128**, 313–323. (doi:10.1017/S0950268801006501)

45. Alexandersen S, Zhang Z, Donaldson AI, Garland AJM. 2003 The Pathogenesis and Diagnosis of Foot-and-Mouth Disease. *J. Comp. Pathol.* **129**, 1–36. (doi:10.1016/S0021-9975(03)00041-0)

46. Liu Y, Yan LM, Wan L, Xiang TX, Le A, Liu JM, Peiris M, Poon LLM, Zhang W. 2020 Viral dynamics in mild and severe cases of COVID-19. *Lancet Infect. Dis.* **20**, 656–657. (doi:10.1016/S1473-3099(20)30232-2)

47. Garland AJ. 1974 The inhibitory activity of secretions in cattle against FMDV. PhD Thesis, University of London.

48. Wang W, Xu Y, Gao R, Lu R, Han K, Wu G, Tan W. 2020 Detection of SARS-CoV-2 in Different Types of Clinical Specimens. *JAMA - J. Am. Med. Assoc.* **323**, 1843–1844. (doi:10.1001/jama.2020.3786)

49. Sellers RF. 1971 Quantitative aspects of the spread of foot and mouth disease. *Vet. Bull.* **41**, 431–439.

50. WHO. In press. Modes of transmission of virus causing COVID-19: implications for IPC precaution recommendations. See https://www.who.int/news-room/commentaries/detail/modes-of-transmission-of-virus-causing-covid-19-implications-for-ipc-precaution-recommendations (accessed on 5 June 2020).

51. Ong SWX, Tan YK, Chia PY, Lee TH, Ng OT, Wong MSY, Marimuthu K. 2020 Air, Surface Environmental, and Personal Protective Equipment Contamination by Severe Acute Respiratory Syndrome Coronavirus 2 (SARS-CoV-2) from a Symptomatic Patient. *JAMA - J. Am. Med. Assoc.* **323**, 1610–1612. (doi:10.1001/jama.2020.3227)

52. McVicar JW, Sutmoller P. 1976 Growth of foot-and-mouth disease virus in the upper respiratory tract of non-immunized, vaccinated, and recovered cattle after intranasal inoculation. *J. Hyg. (Lond).* **76**, 467–481. (doi:10.1017/S0022172400055406)

53. Cottral GE, Patty RE, Gailiunas P, Scott FW. 1965 Sensitivity of cell cultures, cattle, mice, and guinea-pigs for detection of nineteen foot-and-mouth disease viruses. *OIE Bull.* **63**, 1607–1625.

54. Wu Y *et al.* 2020 Prolonged presence of SARS-CoV-2 viral RNA in faecal samples. *Lancet Gastroenterol. Hepatol.* **5**, 434–435. (doi:10.1016/S2468-1253(20)30083-2)

55. Woolhouse MEJ, Haydon DT, Pearson A, Kitching RPP. 1996 Failure of vaccination to prevent outbreaks of foot and mouth disease. *Epidemiol Infect* **116**, 363–371.

56. McCullough KC, De Simone F, Brocchi E, Capucci L, Crowther JR, Kihm U, Simone FDE. 1992 Protective immune response against foot-and-mouth disease. *J Virol* **66**, 1835–1840.

57. Jacofsky D, Jacofsky EM, Jacofsky M. 2020 Understanding Antibody Testing for COVID-19. *J. Arthroplasty* (doi:10.1016/j.arth.2020.04.055)

58. Zhou F *et al.* 2020 Clinical course and risk factors for mortality of adult inpatients with COVID-19 in Wuhan, China: a retrospective cohort study. *Lancet* **395**, 1054–1062. (doi:10.1016/S0140-6736(20)30566-3)

59. Burrows R. 1968 Excretion of foot-and-mouth disease virus prior to the development of lesions. *Vet Rec* **83**, 387–388.

***Figure 2 additional references***

64. UK Gov. 2020 The Health Protection (Coronavirus, Restrictions) (England) (Amendment) (No. 3) Regulations 2020. The National Archives. Available at: http://www.legislation.gov.uk/uksi/2020/558/introduction/made Accessed 15 June 2020.

65. UK Gov Prime Minister’s Office. 2020 PM address to the nation on coronavirus: 10 May 2020. Government Digital Service; 10 May 2020. Available at: https://www.gov.uk/government/speeches/pm-address-to-the-nation-on-coronavirus-10-may-2020. Accessed 7 June 2020.

66. WHO. 2020 Rolling updates on coronavirus disease (COVID-19). Available at: https://www.who.int/emergencies/diseases/novel-coronavirus-2019/events-as-they-happen. Accessed 15 June 2020.

67. UK Gov Department of Health and Social Care. 2020 CMO confirms cases of coronavirus in England. Government Digital Service; 31 January 2020. Available at: https://www.gov.uk/government/news/cmo-confirms-cases-of-coronavirus-in-england. Accessed 7 June 2020.

68. BBC. 2020 Coronavirus: Latest patient was first to be infected in UK; 29 February 2020. Available at: https://www.bbc.com/news/uk-51683428.html. Accessed 7 June 2020.

69. UK Gov. Dept of Health and Social Care. 2020 COVID-19: government announces moving out of contain phase and into delay. Government Digital Service; 12 March 2020. Available at: https://www.gov.uk/government/news/covid-19-government-announces-moving-out-of-contain-phase-and-into-delay. Accessed 7 June 2020.

70. UK Gov Ministry of Defence. 2020 COVID Support Force: the MOD’s contribution to the coronavirus response. Government Digital Service; 23 March 2020. Available at: https://www.gov.uk/guidance/covid-support-force-the-mods-contribution-to-the-coronavirus-res Accessed 7 June 2020.

71. UK Gov Prime Minister’s Office. 2020 PM address to the nation on coronavirus: 23 March 2020. Government Digital Service; 23 March 2020. Available at: https://www.gov.uk/government/speeches/pm-address-to-the-nation-on-coronavirus-23-march-2020 Accessed 7 June 2020.

72. UK Gov Prime Minister’s Office. 2020 Statement from Downing Street: 5 April 2020. Government Digital Service; 5 April 2020 Available at: https://www.gov.uk/government/news/statement-from-downing-street-5-april-2020 Accessed 7 June 2020.

73. The Health Foundation. 2020 COVID-19 policy tracker. A timeline of national policy and health system responses to COVID-19 in England. Available at www.health.org.uk/news-and-comment/charts-and-infographics/covid-19-policy-tracker Accessed 28 June 2020

74. EU Open Data Portal. COVID-19 cases worldwide. Available at: https://www.ecdc.europa.eu/sites/default/files/documents/COVID-19-geographic-disbtribution-worldwide.xlsx. Accessed 16 June 2020.
